# Effect of Endogenous Lipids and Proteins on the Antioxidant, *in vitro* Starch Digestibility, and Pasting Properties of Sorghum Flour

**DOI:** 10.3389/fnut.2021.809330

**Published:** 2022-01-13

**Authors:** Emmanuel Anyachukwu Irondi, Adekemi Esther Adewuyi, Tolulope Muktar Aroyehun

**Affiliations:** Department of Medical Biochemistry and Pharmacology, Kwara State University, Ilorin, Nigeria

**Keywords:** antioxidant activities, endogenous lipids and proteins, pasting properties, sorghum bicolor flour, starch digestibility

## Abstract

This study evaluated the effect of endogenous lipids and proteins on the antioxidants, starch digestibility, and pasting properties of sorghum (*Sorghum bicolor*) flour (SF). Endogenous lipids and/or proteins were removed from different portions of SF to obtain defatted (DF), deproteinized (DP), and defatted and deproteinized (DF-DP) flours. Bioactive constituents (total phenolics, tannins, flavonoids, saponins, and anthocyanins), antioxidant activities [2,2-Azinobis (3-ethyl-benzothiazoline-6-sulfonic acid) radical cation (ABTS^*+^) and 2, 2-Diphenyl-2-picrylhydrazyl radical (DPPH^*^) scavenging activities, reducing power, and Fe^2+^ chelating capacity], starch, amylose, starch hydrolysis index (HI), estimated glycemic index (eGI), and pasting properties of treated and control (untreated) flours were determined. The control flour (SF) had significantly higher (*p* < 0.05) levels of all the bioactive constituents and antioxidant activity tested than the DF, DP, and DF-DP flours, while the DF-DP flour had the least levels of bioactive constituents and antioxidant activity. In contrast, the starch, amylose, HI, and eGI were consistently in the order of DF-DP > DF > DP > control flour (*p* < 0.05). The control flour had the highest (*p* < 0.05) peak viscosity, and the least peak time and pasting temperature, while the DF flour had the highest final viscosity. Therefore, endogenous lipids and proteins contribute to the antioxidant, starch digestibility, and pasting properties of sorghum flour.

## Introduction

Sorghum [*Sorghum bicolor* (L.) Moench] is a major cereal for millions of poor people in semi-arid tropical regions of Africa, Asia, and Latin America (1). It serves as an important source of calorie and protein for many people in the semi-arid tropical regions of the world (2). Sorghum has been applied in several food applications. It is processed into various traditional products, such as *Aceda* (thick porridge) and *Hulu-mur* (non-alcoholic beverages) (3) in different regions of the world, and bakery and supplementary health foods and products (4). The flour does not contain gluten, and for this reason, it is an alternative for people allergic to gluten (5).

In addition to its rich nutritive value, including proteins, starch, B-complex vitamins and minerals in the endosperm, as well as lipids and some fat-soluble vitamins in the germ (6), whole sorghum grain contains high levels of bioactive constituents such as polyphenols. These bioactive constituents confer on sorghum some bioactivities such as antioxidant and enzymes inhibitory activities (7, 8), and health benefits such as reduction in the risk of developing chronic diseases including hypertension, diabetes mellitus, obesity, and cancer (6).

Starches, proteins, and lipids are the major constituents of staple crops, including sorghum, which play vital roles in both human and animal nutrition. The interactions among these three major constituents in a food system have significant implications on the quality and functionality of the cereal-based products (9) and are capable of influencing their physicochemical properties and bioactivities. For instance, the digestion of starch in rice flour was retarded by endogenous lipids and proteins; an effect attributed to the reduction in starch swelling power by the endogenous lipids and proteins (10). Annor et al. (11) also reported that the *in vitro* starch digestibility and estimated glycemic index of kodo millet flour increased significantly after the removal of protein and/or lipid, especially after the removal of lipid. On the other hand, the addition of the protein was found to inhibit the catalytic activity of amylase against starch granules (12). These interactions, in turn, can influence postprandial blood-glucose response (13). Similarly, amylose, the linear component of starch, is known to form single-helical complexes with lipids (14), which have been explored to produce starch products with better qualities for application in different foods (15).

As Zhang and Hamaker (9) noted, the study of the interactions of food ingredients can boost the understanding of their functionalities in real food systems and provide useful information for the food industry. Hence, in view of the importance of sorghum grain as a major source of nutrients and bioactive components, and the potential interactions among its chemical constituents, this study set out to evaluate the effect of endogenous lipids and proteins on the antioxidant, pasting properties, and *in vitro* starch digestibility of sorghum flour.

## Materials and Methods

### Materials

A sample (1 kg) of red sorghum grains was procured from the grains market in Minna, Nigeria. The sample was sorted and ground into flour. The sorghum flour (SF) was packed hermetically in an opaque plastic container, and stored at 4°C during analysis. Analytical grades of all the chemicals and reagents were used in all the experiments.

### Defatting and Deproteinization of Sample

Endogenous lipids and/or proteins were removed from different portions (500 g) of SF to obtain defatted (DF), deproteinized (DP), and defatted and deproteinized (DF-DP) flours, according to the method described by Annor et al. (11) with slight modifications. Defatting was carried out by blending 100 g of SF with 500 ml of petroleum ether, and stirring continuously at room temperature for 4 h. Thereafter, the mixture was filtered and the solid residue was collected. The process was repeated two more times on the solid residue, to obtain SF without lipids. Deproteinization of SF was carried out by alkaline protease hydrolysis. A portion of 800 ml of alkaline protease solution (120 U/ml) in 0.02 M carbonate buffer (pH 9.0) was added to 100 g of SF in a beaker. The suspension was placed in a water bath set at 45°C for 4 h with continuous stirring for hydrolysis to take place, and centrifuged at 4,000 rpm for 10 min. The solid residue was subjected to another round of hydrolysis with alkaline proteases by the same procedure, after which the resulting solid residue was washed to neutral pH with distilled water. Defatted-deproteinized SF was prepared by sequentially defatting and deproteinizing 100 g of SF by following the methods described above. Finally, defatted, deproteinized, and defatted-deproteinized SF samples were oven-dried at 45°C, and stored in air-tight sample containers.

### Preparation of Methanolic Extracts

Extraction of flour samples for bioactive constituents and antioxidant assays was carried out as described by Engida et al. (16). Two grams each of the control (untreated) (SF) and treated (DF, DP, DF-DP) flours were extracted with methanol (20 ml) overnight. Thereafter, the mixture was centrifuged (4,000 rpm for 5 min). The resulting supernatant was collected, concentrated in a rotary evaporator at 45°C, and subsequently reconstituted with 6 ml methanol.

### Determination of Bioactive Constituent (Total Phenolics, Tannins, Flavonoids, Saponins, and Anthocyanins) Contents

Total phenolics level of the flour extracts was determined as per Folin–Ciocalteu method described by Singleton et al. (17) and expressed as gallic acid equivalent in milligram per gram sample (GAE mg/g). Level of tannins was quantified as per the method described by Amorim et al. (18) and expressed as tannic acid equivalent in milligram per gram sample (TAE mg/g). Total flavonoids level was determined according to aluminum chloride method described by Meda et al. (19) and expressed as quercetin equivalent in milligram per gram sample (QE mg/g). Total saponins level was determined as per the method described by Makkar et al. (20) and expressed as diosgenin equivalent in milligram per gram sample (DE mg/g). Total anthocyanins level was quantified by pH differential method as described by Finocchiaro et al. (21) and expressed as cyanidin glucoside equivalent in milligram per gram sample (CGE mg/g).

### ABTS^*+^ Scavenging Assay

ABTS^*+^ scavenging assay was carried out as described by Re et al. (22). To prepare ABTS^*+^ reagent, equal volumes of aqueous solutions of ABTS^*+^ (7 mM) and K_2_S_2_O_8_ (2.45 mM) were mixed and incubated in the dark at room temperature for 16 h. Afterward, the absorbance of the reagent was adjusted to 0.7 ± 0.02 at 734 nm using 95% ethanol. For the assay, a mixture of 2.0 ml of the ABTS^*+^ reagent and 0.2 ml of each extract was incubated at room temperature for 15 min. Afterward, the absorbance was read at 734 nm in a UV-Vis spectrophotometer. ABTS^*+^ scavenging ability of the extracts was calculated using Trolox calibration curve, and expressed as Trolox equivalent antioxidant capacity in micromole per gram sample dry weight (TEAC μmol/g DW).

### DPPH^*^ Scavenging Assay

DPPH^*^ scavenging assay was conducted as described by Cervato et al. (23), using ascorbic acid as a reference antioxidant. A mixture of 1.0 ml of different concentrations (0.15, 0.30, 0.45, and 0.60 mg/ml) of the extracts and 3.0 ml of 60 μM DPPH^*^ solution was incubated at room temperature for 30 min. Thereafter, the absorbance was read at 517 nm in a UV-Vis spectrophotometer, and the DPPH^*^ scavenging capacity of the extracts, expressed as half-maximal scavenging concentration (SC_50_), was calculated.

### Reducing Power Assay

Reducing power assay was carried out as described by Oyaizu (24). A mixture of 2.5 ml of extract, 2.5 ml of 200 mM sodium phosphate buffer (pH 6.6), and 2.5 ml of 1% potassium ferricyanide was incubated at 50°C for 20 min. Next, 2.5 ml of 10% trichloroacetic acid was added. The mixture was then divided into aliquots of 2.5 ml in different test tubes, and each portion was diluted with 2.5 ml of distilled H_2_O. Afterwards, 1 ml of 0.1% ferric chloride was added to each tube and the absorbance was read at 700 nm. Reducing power of the extracts was calculated using gallic acid calibration curve, and expressed as gallic acid equivalent in milligram per gram sample dry weight (GAE mg/g DW).

### Iron (II) (Fe^2+^) Chelating Assay

Iron (II) **(**Fe^2+^) chelating assay was conducted as reported by Puntel et al. (25), using ascorbic acid as a reference antioxidant. A mixture of 168 μl 0.1 M Tris-HCl (pH 7.4), 218 μl normal saline, and different concentrations of the extracts (0.30, 0.60, 0.90, and 1.20 mg/ml) and 150 μl of freshly prepared FeSO_4_ (500 μM) was incubated at room temperature for 5 min. Afterwards, 13 μl of 0.25% 1,10-phenanthroline was added, and the absorbance was read at 510 nm in a UV-Vis spectrophotometer. Fe^2+^ chelating capacity of the extracts was calculated and expressed as half-maximal scavenging concentration (SC_50_) in mg/mL.

### Determination of Starch Content

Starch content of the flour samples was quantified as described by Onitilo et al. (26) with minor modification. A mixture of 0.02 g of sample, 1 ml of 80% ethanol, 2 ml of distilled water, and 10 ml of hot 80% ethanol was centrifuged at 2,000 rpm for 10 min. The solid residue was hydrolyzed with 7.5 ml of concentrated perchloric acid for 1 h. Afterward, the hydrolysate was diluted to 25 ml with distilled water and filtered through a Whatman (No. 2) filter paper. Next, 0.05 ml of the filtrate, 0.5 ml of 5% phenol solution, and 2.5 ml of H_2_SO_4_ were mixed in a test tube, and allowed to cool to room temperature, after which the absorbance was read at 490 nm. Starch content of sample was calculated using a D-glucose calibration curve.

### Determination of Amylose and Amylopectin Contents

Amylose content of the flour samples was determined as described by Juliano et al. (27). A mixture of 100 mg of flour sample, 1 ml of 95% ethanol, and 9.2 ml of 1 M NaOH was heated at 100°C in a water bath for 10 min to gelatinize the sample. After cooling to room temperature, 0.05 ml of the gelatinized sample was diluted with 0.45 ml of distilled water and mixed with 0.1 ml of acetic acid solution (1 N), 0.2 ml of iodine solution (0.2% I_2_ in 2% KI), and 9.2 ml of distilled water. The mixture was incubated for 20 min at room temperature, following which the absorbance was read at 620 nm in a UV-Vis spectrophotometer. Amylose content of the samples was computed using amylose standard.

Amylopectin level of the samples was calculated as per the formula reported by Juan et al. (28) as follows:


$$\begin{array}{l} \text{Amylopectin }\left(\text{\%}\right)=100-\text{amylose }\left(\text{\%}\right). \end{array}$$

### Determination of *in vitro* Starch Digestibility and Estimated Glycemic Index

The *in vitro* starch digestibility rate and hydrolysis index (HI) of the flour samples were determined as per the method of Goni et al. (29). The rate of starch digestion was expressed as the percentage of starch hydrolyzed per unit time. Glucose (50 mg) was used as the standard.


$$\begin{array}{l} \text{Hydrolysis Index }\left(\text{\%}\right)=\frac{\text{AUC }\left(\text{sample}\right)\times 100}{\text{AUC }\left(\text{ref}\right)} \end{array}$$

where AUC (sample) and AUC (ref) are the areas under the hydrolysis curves of the flour sample and reference/standard carbohydrate (50 g glucose), respectively.

The estimated glycemic index (eGI) of samples was calculated using the following formula: eGI = 39.71 + 0.549HI; where GI = Glycemic index (%) and HI = Hydrolysis index (%).

### Determination of Pasting Properties

The pasting properties of the flour samples were analyzed using a Rapid Visco Analyzer (RVA) (RVA-4, Perten Scientific, Springfield, IL) (30). The RVA was connected to a personal computer (PC) system installed with Thermocline software. The flour sample (3 g) was dispersed in 25 ml of distilled water in a canister and loaded on the RVA. Pasting properties of the flour, including peak, trough, breakdown, final and set-back viscosities [expressed in Rapid Visco Analyzer Units (RVU)], peak time (in minutes) and pasting temperature (in °C), were then read on the PC system with the aid of the Thermocline software.

### Data Analysis

Results of three independent determinations were expressed as mean ± SD. The mean values were subjected to one-way ANOVA, and the mean values of different treatments were compared using Duncan's multiple range test at *p* < 0.05. The 17th version of Statistical Package for Social Science (SPSS) software was used for data analysis.

## Results and Discussion

### Bioactive Constituents of Control and Treated Sorghum Flours

The levels of bioactive constituents of the control, defatted (DF), deproteinized (DP), and defatted and deproteinized (DF-DP) sorghum flour samples are presented in Table 1. Consistently, the levels of all the bioactive constituents varied significantly (*p* < 0.05), such that the control > DF > DP > DF-DP flour. The total phenolics, tannins, total flavonoids, saponins, and anthocyanins contents of the flours ranged from 1.54 ± 0.06 to 7.48 ± 0.51 mg GAE/g; 1.69 ± 0.08 to 8.23 ± 0.62 mg TAE/g; from 0.97 ± 0.04 to 4.69 ± 0.04 mg QE/g; from 0.77 ± 0.03 to 3.74 ± 0.04 mg DE/g; from 1.93 ± 0.06 to 9.35 ± 0.60 CGE mg/g, respectively, in DF-DP and control flours.

**Table 1:** Bioactive constituents of control, defatted, deproteinized, and defatted-deproteinized sorghum flour.

**Flour**	**Total phenolics (GAE mg/g)**	**Tannins (TAE mg/g)**	**Total flavonoids (QE mg/g)**	**Total saponins (DE mg/g)**	**Total anthocyanins (CGE mg/g)**
Control	7.48 ± 0.51^a^	8.23 ± 0.62^a^	4.69 ± 0.04^a^	3.74 ± 0.04^a^	9.35 ± 0.60^a^
Defatted	3.67 ± 0.02^b^	4.03 ± 0.23^b^	2.30 ± 0.08^b^	1.83 ± 0.01^b^	4.58 ± 0.30^b^
Deproteinized	2.24 ± 0.13^c^	2.47 ± 0.11^c^	1.41 ± 0.01^c^	1.12 ± 0.01^c^	2.80 ± 0.11^c^
Defatted-deproteinized	1.54 ± 0.06^d^	1.69 ± 0.08^d^	0.97 ± 0.04^d^	0.77 ± 0.03^d^	1.93 ± 0.06^d^

*Results are means ± SD of triplicate determinations. Along the same row, values having different superscript letters vary significantly (p < 0.05). GAE, gallic acid equivalent; TAE, tannic acid equivalent; QE, quercetin equivalent; DE, diosgenin equivalent; CGE, cyanidin glucoside equivalents*.

The total phenolics (7.48 ± 0.51 mg GAE/g), tannins (8.23 ± 0.62 mg TAE/g), and total flavonoids (4.69 ± 0.04 mg QE/g) contents of the control SF were lower than the levels of these bioactive constituents (total phenolics, 8.33 ± 0.55 GAE mg/g; condensed tannins, 8.63 ± 0.89 mg catechin equivalent/g; total flavonoids, 6.59 ± 0.40 mg catechin equivalent/g) previously reported for whole sorghum flour (31). The lower concentrations of the bioactive constituents observed in this study, relative to the levels reported by Moraes et al. (31), could be attributed to differences in genotypic, environmental, and sample extraction methods (32). Total phenolics, tannins, total flavonoids, saponins, and anthocyanins possess antioxidant activity (21, 33), among other health benefits. These bioactive constituents exhibit their antioxidant activity by different mechanisms, including scavenging of free radicals, prevention of chain initiation and continued hydrogen abstraction, reducing capacity, binding of transition metal ion catalysts, and decomposition of peroxides (34). In addition to their antioxidant activity, these bioactive constituents also possess other important health benefits. The polyphenolic compounds (tannins and flavonoids), for instance, are prominent for their anti-inflammatory, anti-cancer, anti-microbial, anti-Alzheimer's, anti-allergic, anti-diabetic, and anti-hypertensive activities among other health benefits (8, 35). Furthermore, the antioxidant properties of these bioactive constituents, especially the phenolic compounds, prevent the oxidative degradation of some nutrients that are highly susceptible to oxidation, such as vitamins and unsaturated fatty acids (36), and retard the formation of some toxic oxidative products, thereby maintaining the nutritional quality and extending the shelf-life of food products (35).

### Antioxidant Activity of Control and Treated Sorghum Flours

The results of antioxidant activity of the control, DF, DP, and DF-DP sorghum flour samples, as ascertained using four different assays (ABTS^*+^ and DPPH^*^ scavenging, Fe^2+^ chelation, and reducing power assays) are presented in Table 2. The antioxidant activity of the flour varied significantly (*p* < 0.05) due to the removal of lipids and/or proteins, whereas the ABTS^*+^ scavenging ability and reducing power were in the order of control flour > DF > DP > DF-DP flour. The DPPH^*^ and Fe^2+^ chelation SC_50_ values had a reverse order of DF-DP > DP > DF > control flour. Taken together, these trends indicate that the control flour, containing the endogenous lipids and proteins, had the strongest antioxidant activity, while the DF-DP flour had the weakest antioxidant activity. This notwithstanding, ascorbic acid (a reference antioxidant compound) had stronger DPPH^*^ scavenging and Fe^2+^ chelation abilities than the control flour, as indicated by its lower DPPH^*^ and Fe^2+^ chelation SC_50_ values (6.08 ± 0.57 and 8.89 ± 0.91 μg/ml, respectively). However, the DPPH^*^ scavenging ability of the control flour (SC_50_: 8 ± 0.98 μg/ml) was stronger than the DPPH^*^ scavenging ability earlier reported (SC_50_: 12.04 ± 0.85 μg/ml) for *S. bicolor*, as a lower SC_50_ value represents a stronger scavenging activity (8). Similarly, the ABTS^*+^ scavenging activity of the control flour in this study (7.52 ± 0.45 mmol TEAC/g) is much higher than the range of ABTS^*+^ scavenging activity (61.6 – 125 μmol TE/g, equivalent to 0.062 – 0.125 mmol/g) that Awika et al. (37) reported for seven different varieties of *S. bicolor*. As earlier stated, these variations could have stemmed from differences in the *S. bicolor* genotype, environmental factors, and the methods of sample extraction adopted (32).

**Table 2:** Antioxidant activity of control, defatted, deproteinized, and defatted-deproteinized sorghum flour.

**Flour**	ABTS^*+^ scavenging ability (mmol TEAC/g)	DPPH* SC_**50**_ (μg/mL)	**Fe^**2+**^ chelation SC_**50**_ (μg/mL)**	**Reducing power** **(mg GAE/g)**
Control	7.52 ± 0.45^a^	8 ± 0.98^d^	11.34 ± 0.92^d^	68.38 ± 1.63^a^
Defatted	5.26 ± 0.23^b^	11.81 ± 0.87^c^	15.81 ± 0.83^c^	65.36 ± 1.21^b^
Deproteinized	4.02 ± 0.19^c^	13.49.58 ± 0.74^b^	17.58 ± 1.11^b^	61.81 ± 1.06^c^
Defatted-deproteinized	3.23 ± 0.13^d^	16.37 ± 1.03^a^	19.64 ± 1.42^a^	56.29 ± 1.51^d^
Ascorbic acid	-	6.08 ± 0.57^e^	8.89 ± 0.91^e^	-

Results are means ± SD of triplicate determinations. Along the same column, values having different superscript letters vary significantly (p < 0.05). SC_50_: extract concentration that scavenged 50% of DPPH^*^; TEAC, trolox equivalent antioxidant capacity; GAE, gallic acid equivalent.

The decrease in the antioxidant activity of the DF, DP, and DF-DP flours could be due to the reduction in the bioactive constituents occasioned by the defatting, deproteinization, and defatting and deproteinization of the flour. These bioactive constituents, which are known to exist either in bound or free states within the food matrix, are the determinants of the antioxidant activity of the sample (38). For instance, phenolic compounds exist mainly as glycosides linked to various moieties of sugar or as other complexes linked to lipids, carbohydrates, organic acids, amines, and other phenols (39). It is also possible that the endogenous proteins contributed to the antioxidant activity of the sorghum flour, as bioactive peptides from different plants were reported to display antioxidant activity (40).

The free radicals (ABTS^*+^ and DPPH^+^)-scavenging activity of the flour is indicative that it could help prevent and/or ameliorate oxidative stress when ingested as food. It is important to recall that cellular oxidative stress sets in when the oxidant burden of the cell outweighs its antioxidant defense system. Oxidative stress, in turn, precipitates some non-communicable diseases such as gout, obesity, and diabetes mellitus (41).

Furthermore, in biological systems, iron is the most abundant transition metal ion that strongly catalyzes the production of free radicals and reactive oxygen species (ROS), which can attack biomolecules, such as and lipid, DNA, and protein. Thus, the ability of the control sorghum flour to chelate Fe^2+^ suggests that it can retard and/or inhibit Fe^2+^-catalyzed production of free radicals and ROS, thereby preventing the oxidative damage of biomolecules (42). In addition to the protective effects of the antioxidant activity of the control flour on the cellular biomolecules, it also helps in preserving some endogenous nutrients from oxidative degradation (36).

### Starch, Amylose, and Amylopectin Levels, and Starch Hydrolysis and Estimated Glycemic Index of Control and Treated Sorghum Flours

Table 3 presents the starch, amylose, amylopectin contents, amylose/amylopectin ratios, starch hydrolysis index (HI), and estimated glycemic index (eGI) of the control, DF, DP, and DF-DP sorghum flours. The starch and amylose contents were consistently and significantly (*p* < 0.05) higher in the DF-DP flour, followed by the DF, DP, and control flours. In contrast, the control flour had the highest (*p* < 0.05) amylopectin level followed by DP, DF, and DF-DP flours. The amylose/amylopectin ratios of the DF and DF-DP flours were comparable (*p* > 0.05), but both were significantly higher than those of the control and DP flours. There were significant (*p* < 0.05) and consistent reductions in the HI and eGI of the flour as a result of defatting, deproteinization, and defatting and deproteinization, such that DF-DP > DF > DP > control flour.

**Table 3:** Starch, amylose, amylopectin, starch hydrolysis index (HI), and estimated glycemic index (eGI) of control, defatted, deproteinized, and defatted-deproteinized sorghum flour.

**Flour**	**Starch (%)**	**Amylose (%)**	**Amylopectin (%)**	**Amylose/amylopectin**	**HI (%)**	**eGI (%)**
Control	47.72 ± 0.31^d^	22.67 ± 0.22^d^	77.34 ± 0.22^a^	0.30 ± 0.01^b^	65.53 ± 0.64^a^	75.69 ± 0.35^a^
Defatted	59.58 ± 0.33^b^	26.74 ± 0.30^b^	73.26 ± 0.30^c^	0.37 ± 0.02^a^	72.10 ± 0.58^c^	79.30 ± 0.32^c^
Deproteinized	52.99 ± 0.31^c^	24.01 ± 0.52^c^	75.99 ± 0.52^b^	0.32 ± 0.01^b^	68.72 ± 0.71^b^	77.44 ± 0.39^b^
Defatted- deproteinized	70.99 ± 0.34^a^	27.83 ± 0.22^a^	72.18 ± 0.22^d^	0.39 ± 0.03^a^	80.83 ± 0.25^d^	84.09 ± 0.13^d^

*Results are means ± SD of triplicate determinations. Along the same column, values having different superscript letters vary significantly (p < 0.05)*.

Starch, comprising amylose and amylopectin and morphologically occurring as water-insoluble semi-crystalline granules, has a wide range of applications in food products due to its peculiar thickening, gelling, and stabilizing qualities (43). In both the control and treated flours in this study, the amylopectin level was consistently higher than the level of amylose, and this corroborates previous reports that in most starches, amylopectin is more abundant than amylose (44). The composition of amylose and amylopectin in starchy flours impacts their functional attributes, which in turn determines their applications in the development of food products and industrial uses. Also, they have an influence on the glycemic index (GI) of the starchy flour, such that a higher amylopectin and a lower amylose composition translate to a higher GI, an effect attributed to the relative ease of α-amylase-catalyzed hydrolysis of amylopectin and amylose in the human duodenum (45).

Contrary to the expectation that a lower level of amylose in the control flour would translate to a higher rate of its starch hydrolysis (digestibility) by α-amylase, thereby releasing more monosaccharides (such as glucose and fructose), with a concomitant higher GI (46), the HI and eGI proved otherwise. Obviously, the control flour had the least (*p* < 0.05) HI and eGI, relative to the DF, DP, and DF-DP flours (Table 3). This buttresses the impact of extrinsic factors, including food matrix effect (in this case, the endogenous lipids and proteins) on the HI and eGI (10, 47) of the sorghum flour. For instance, it had been previously demonstrated that lipid lowered starch digestibility by forming a physical complex (amylose–lipid complex) with the starch molecules (48). Similarly, López–Barón et al. (47) reported that plant proteins caused a reduction in the *in vitro* digestibility of wheat starch. This reduction was attributed to the propensity of proteins to surround the granules of starch, thereby limiting the access of starch-digesting enzymes (α-amylase and α-glucosidase) to the starch granules (10, 11). Taken together, the reduction in the *in vitro* starch digestibility of the control sorghum flour due to the presence of endogenous lipids and proteins suggests that the endogenous lipids and proteins could lower the glycemic index of the sorghum flour.

Furthermore, significant differences (*p* < 0.05) were observed in the HI and eGI of the treated flours, such that the DF-DP flour had the highest HI and eGI, followed by the DF flour, and lastly the DP flour. This indicates that the removal of the endogenous lipids enhanced the *in vitro* digestibility of the sorghum starch more than the removal of endogenous proteins. However, this trend is different from the trend observed by Ye et al. (10) in rice flour, in which the *in vitro* starch digestibility of the DF-DP flour was followed by that of the DP flour and finally by that of the DF flour. This difference in trend could be due to the possible variation in the matrix of the rice flour investigated by Ye et al. (10) and the sorghum flour evaluated in this study. Amylose displays resistance to amylase-catalyzed hydrolysis when complexed with other compounds such as lipids and proteins (15). Thus, the amylose–protein complex in sorghum may have been more resistant to amylase-catalyzed hydrolysis than the amylose–fat complex. According to Chen et al. (15), protein (soy protein) decreased the enzymatic digestion of corn starch more than oil (corn oil). The possible reasons for this observation are 2-fold. First, proteins can situate on the surface of starch, encapsulate the starch granules, and consequently, prevent and/or retard starch hydrolysis. Second, the different hydrophilic groups present in proteins, including -NH_2_, -COOH, -SH, and -OH, can bind with starch through hydrogen bond, causing an adhesion on the protein. The resultant viscosity from the protein adhesion could retard the propensity of starch to enzyme hydrolysis.

### Pasting Properties of Control and Treated Sorghum Flours

**Figure 1:**
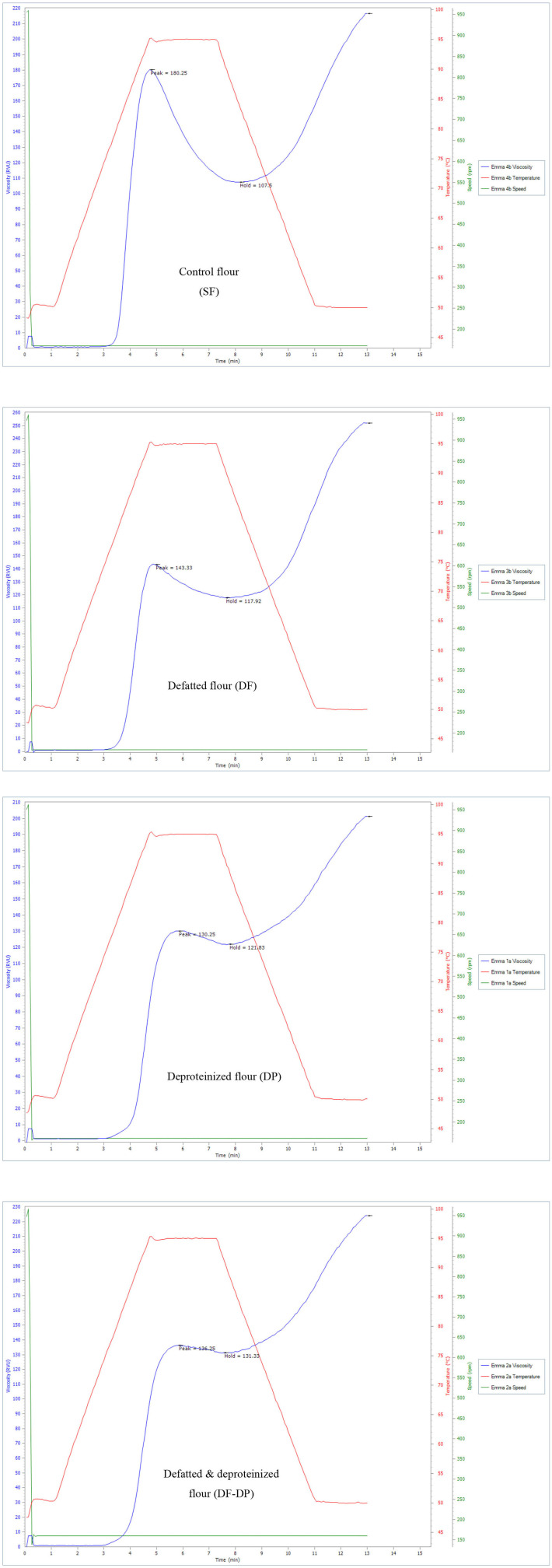
Representative amylographs of control (SF), defatted (DF), deproteinized (DP), and defatted and deproteinized (DF-DP) red sorghum flour samples.

The pasting properties of the flour were determined to provide insight into how the endogenous lipids and/or proteins could influence the potential food and industrial applications of the flour. The representative amylographs of the flours are shown in Figure 1. The process of starch gelatinization and pasting involve the following order of events: starch granule swelling, leaching of amylose, a three-dimensional starch network formation of the leached amylose, and finally, interactions between granule remnants and the leached amylose (43). As presented in Table 4, the endogenous lipids and/or proteins had a significant effect (*p* < 0.05) on the pasting profiles of the flour. The control flour had the highest peak and breakdown viscosities (180.42 ± 0.23 and 71.96 ± 1.12 RVU, respectively), and the lowest peak time and pasting temperature (4.73 ± 0.01 min and 80.83 ± 0.04°C, respectively). DF flour had the highest final and setback viscosities (250.46 ± 1.82 and 133.67 ± 0.23 RVU, respectively), while DP flour had the lowest final and setback viscosities (204.46 ± 4.54 and 83.63 ± 5.95 RVU, respectively). The result further revealed that the DF-DP flour had the highest trough viscosity (128.04 ± 4.65 RVU) and peak time (9.00 ± 0.01 min). In addition, the pasting temperature of the DF-DP (89.25 ± 1.77°C) and DP (90.50 ± 0.07°C) flours were comparable (*p* > 0.05), but these were both significantly higher than those of the DF (83.63 ± 0.53°C) and control (80.83 ± 0.04°C) flours. The lower peak time and pasting temperature of the control flour, relative to the treated flours, could be directly attributed to the removal of the endogenous lipids and proteins from the treated flours. As earlier suggested by Ye et al. (10), the presence of endogenous lipids and proteins in the control flour could have restricted the propensity of starch granule swelling during heating in water, resulting in lower peak time and pasting temperature.

**Table 4:** Pasting properties of control, defatted, deproteinized, and defatted-deproteinized sorghum flour samples.

**Sample**	**Peak viscosity (RVU)**	**Trough viscosity (RVU)**	**Breakdown viscosity (RVU)**	**Final viscosity (RVU)**	**Set-back viscosity (RVU)**	**Peak time (min)**	**Pasting temp (**°**C)**
Control	180.42 ± 0.23^a^	108.46 ± 1.36^c^	71.96 ± 1.12^a^	218.67 ± 3.06^b^	110.21 ± 1.71^b^	4.73 ± 0.01^c^	80.83 ± 0.04^c^
Defatted	141.54 ± 2.53^b^	116.80 ± 1.59^b^	24.75 ± 0.95^b^	250.46 ± 1.82^a^	133.67 ± 0.23^a^	4.80 ± 0.10^c^	83.63 ± 0.53^b^
Deproteinized	130.79 ± 0.76^c^	120.83 ± 1.41^b^	9.96 ± 2.18^c^	204.46 ± 4.54^b^	83.63 ± 5.95^c^	5.74 ± 0.09^b^	90.50 ± 0.07^a^
Defatted- deproteinized	133.46 ± 3.95^c^	128.04 ± 4.65^a^	5.42 ± 0.71^d^	215.33 ± 12.37^b^	87.29 ± 7.72^c^	9.00 ± 0.01^a^	89.25 ± 1.77^a^

*Results are means ± SD of replicate determinations. Along the same column, values having different superscript letters vary significantly (p < 0.05)*.

Expectedly, the control flour, with the lowest amylose content (Table 3), had the highest peak viscosity (Table 4), in line with earlier reports (45). Peak viscosity indicates the capacity of a flour sample to bind water (49), and represents the propensity of starch granules to swell freely prior to their physical breakdown (45). Previous studies showed that flour with low amylose level easily swelled, due to a weaker binding force in the starch granule, which during heating displayed increased viscosity at a lower temperature (50). Apart from the constituents of starch (amylose and amylopectin), endogenous lipids and proteins also impact the rheological qualities of cereal flours by restricting the expansion of starch granules during gelatinization and decelerating the retrogradation of amylopectin (51). Furthermore, Souare et al. (52) reported that endogenous proteins in food plants increased food viscosity. This could account for why the control flour, containing both the endogenous proteins and lipids, had the highest peak viscosity.

As presented in Table 4, DF had the highest final viscosity. Final viscosity, indicating the stability of starch upon cooking and its paste resistance to shear force while stirring, stands out as the most commonly used pasting property to determine the end-use quality of a particular starch or flour. Hence, the higher final viscosity displayed by the DF suggests that it may possess a better end-use quality than the control and the DP flours (53).

## Conclusions

The removal of endogenous lipids and/or proteins led to a decrease in the levels of all the bioactive constituents (total phenolics, tannins, flavonoids, saponins, and anthocyanins) and antioxidant activity (ABTS^*+^ and DPPH^*^ scavenging activities, reducing power, and Fe^2+^ chelating capacity) of the sorghum flour. Contrarily, the starch, amylose, and *in vitro* starch digestibility (HI and eGI) of the flour increased as a result of the removal of endogenous lipids and/or proteins. The increase in the *in vitro* starch digestibility of sorghum flour upon the removal of endogenous lipids and proteins suggests that the presence of endogenous lipids and proteins may lower the glycemic index of sorghum flour. The control flour had the highest peak and breakdown viscosities, while the DF flour had the highest final viscosity. Therefore, endogenous lipids and proteins contribute to the antioxidant, starch digestibility, and pasting properties of sorghum flour.

## Data Availability Statement

The raw data supporting the conclusions of this article will be made available by the authors, without undue reservation.

## Author Contributions

EI conceptualized the study, supervised the analyses, wrote the original draft, and edited the final draft. AA contributed in conceptualizing the study, analyzed the samples, and collated the data. TA contributed in the methodology, samples and data analysis, and writing of the original draft. All authors contributed to the article and approved the submitted version.

## Conflict of Interest

The authors declare that the research was conducted in the absence of any commercial or financial relationships that could be construed as a potential conflict of interest.

## Publisher's Note

All claims expressed in this article are solely those of the authors and do not necessarily represent those of their affiliated organizations, or those of the publisher, the editors and the reviewers. Any product that may be evaluated in this article, or claim that may be made by its manufacturer, is not guaranteed or endorsed by the publisher.

## References

[1] Correia I Nunes A Barros AS Delgadillo I. Comparison of the effects induced by different processing methods on sorghum proteins. J Cereal Sci. (2010) 51:146–51. 10.1016/j.jcs.2009.11.005

[2] Belton PS Taylor JRN. Sorghum and millets: protein sources for Africa. Trends Food Sci Technol. (2004) 15:94–8. 10.1016/j.tifs.2003.09.002

[3] Hassani A Zarnkow M Becker T. Influence of malting conditions on sorghum (*Sorghum bicolor* (L.) Moench) as a raw material for fermented beverages. Food Sci Technol Int. (2014) 20:453–63. 10.1177/1082013213490710 23751551

[4] Singh A Sharma S Singh B. Effect of germination time and temperature on the functionality and protein solubility of sorghum flour. J Cereal Sci. (2017) 76:131–9. 10.1016/j.jcs.2017.06.003

[5] Pontieri P Mamone G De Caro S Tuinstra MR Roemer E Okot J. Sorghum, a healthy and gluten-free food for celiac patients as demonstrated by genome, biochemical and immunochemical analyses. J Agric Food Chem. (2013) 61:2565–71. 10.1021/jf304882k 23432128

[6] Cardoso LM Montini AT Pinheiro SS. Pinheiro-Sant'Ana HM, Martino HSD, Moreira AVB. Effects of processing with dry heat and wet heat on the antioxidant profile of sorghum. Food Chem. (2014) 152:210–7. 10.1016/j.foodchem.2013.11.106 24444928

[7] Wu L Huang Z Qin P Ren G. Effects of processing on phytochemical profiles and biological activities for production of sorghum tea. Food Res Int. (2013) 53:678–85. 10.1016/j.foodres.2012.07.062

[8] Irondi EA Adegoke BM Effion ES Oyewo SO Alamu EO Boligon AA. Enzymes inhibitory property, antioxidant activity and phenolics profile of raw and roasted red sorghum grains *in vitro*. Food Sci Hum Wellness. (2019) 8:142–8. 10.1016/j.fshw.2019.03.012

[9] Zhang G Hamaker BR. A three component interaction among starch, protein, and free fatty acids revealed by pasting profiles. J Agric Food Chem. (2003) 51:2797–800. 10.1021/jf0300341 12696975

[10] Ye J Hu X Luo S McClements DJ Liang L Liu C. Effect of endogenous proteins and lipids on starch digestibility in rice flour. Food Res Int. (2018) 106:404–9. 10.1016/j.foodres.2018.01.008 29579941

[11] Annor GA Marcone M Bertoft E Seetharaman K. *In vitro* starch digestibility and expected glycemic nidex of Kodo millet (*Paspalum scrobiculatum*) as affected by starch–protein–lipid interactions. Cereal Chem. (2013) 90:211–7. 10.1094/CCHEM-06-12-0074-R30812563

[12] Bhattarai RR Dhital S Gidley MJ. Interactions among macronutrients in wheat flour determine their enzymic susceptibility. Food Hydrocoll. (2016) 61:415–25. 10.1016/j.foodhyd.2016.05.026

[13] Shah A Zhang G Hamaker BR Campanella OH. Rheological properties of a soluble self-assembled complex from starch, protein and free fatty acids. J Food Engr. (2011) 105:444–52. 10.1016/j.jfoodeng.2011.02.048

[14] Putseys J Lamberts L Delcour J. Amylose-inclusion complexes: Formation, identity and physico-chemical properties. J Cereal Sci. (2010) 51:238–47. 10.1016/j.jcs.2010.01.011

[15] Chen X He XW Zhang B Fu X Jane J Huang Q. Effects of adding corn oil and soy protein to corn starch on the physicochemical and digestive properties of the starch. Int J Biol Macromol. (2017) 104(Part A):481–6. 10.1016/j.ijbiomac.2017.06.024 28606841

[16] Engida AM Kasim NS Tsigie YA Ismadji S Huynh LH Ju Y. Extraction, identification and quantitative HPLC analysis of flavonoids from sarang semut (*Myrmecodia pendan*). Indus Crops Prod. (2013) 41:392–6. 10.1016/j.indcrop.2012.04.043

[17] Singleton VL Orthofer R Lamuela-Raventos RM. Analysis of total phenols and other oxidation substrates and antioxidants by means of Folin–Ciocalteau's reagent. Methods Enzymol. (1999) 299:152–78. 10.1016/S0076-6879(99)99017-1

[18] Amorim ELC Nascimento JE Monteiro JM Sobrinho P Araujo TAS Albuquerque UAP. Simple and accurate procedure for the determination of tannins and flavonoid levels and some applications in ethnobotany and ethnopharmacology. Funct Ecosyst Communities. (2008) 2:88–94.

[19] Meda A Lamien CE Romito M Millogo J Nacoulma OG. Determination of the total phenolic, flavonoid and proline contents in Burkina Fasan honey, as well as their radical scavenging activity. Food Chem. (2005) 91:571–7. 10.1016/j.foodchem.2004.10.006

[20] Makkar HPS Siddhuraju P Becker K. Plant Secondary Metabolites. Totowa, NJ, USA: Humana Press Inc. (2007). 10.1007/978-1-59745-425-4_1 19238775

[21] Finocchiaro F Ferrari B Gianinetti A. A study of biodiversity of flavonoid content in the rice caryopsis evidencing simultaneous accumulation of anthocyanins and proanthocyanidins in a black-grained genotype. J Cereal Sci. (2010) 51:28–34. 10.1016/j.jcs.2009.09.003

[22] Re R Pellegrini N Proteggente A Pannala A Yang M Rice-Evans C. Antioxidant activity applying an improved ABTS radical cation decolorization assay. Free Rad Biol Med. (1999) 26:1231–7. 10.1016/S0891-5849(98)00315-3 10381194

[23] Cervato G Carabelli M Gervasio S Cittera A Cazzola R Cestaro B. Antioxidant properties of oregano (*Origanum vulgare*) leaf extracts. J Food Biochem. (2000) 24:453–65. 10.1111/j.1745-4514.2000.tb00715.x29729962

[24] Oyaizu M. Studies on products of browning reaction: antioxidative activity of products of browning reaction prepared from glucosamine. Jpn J Nutr. (1986) 44:307–15. 10.5264/eiyogakuzashi.44.307

[25] Puntel RL Nogueira CW Rocha JBT. Krebs cycle intermediates modulate thiobarbituric reactive species (TBARS) production in Rat Brain *in vitro*. Neurochem Res. (2005) 30:225–35. 10.1007/s11064-004-2445-7 15895826

[26] Onitilo AA Sanni LO Daniel I Maziya-Dixon B. Physicochemical and functional properties of native starches from cassava varieties in Southwest Nigeria. J Food Agric Environ. (2007) 5:108–14. 10.1080/10942910601048994

[27] Juliano BO Perez CM Blakeney B. International Cooperative testing on the amylose content of milled rice. Starch. (1981) 33:157–62. 10.1002/star.1981033050425855820

[28] Juan G Luis A David B. Isolation and molecular characterization of Makal (*Xanthosoma yucatanensis*) starch. Starch. (2006) 58:300–7. 10.1002/star.20050045125855820

[29] Goni L Garcia-Alonso A Saura-Calixto FA. Starch hydrolysis procedure to estimate glycemic index. Nutr Res. (1997) 17:427–37. 10.1016/S0271-5317(97)00010-928372189

[30] Deffenbaugh LB Walker CE. Comparison of starch pasting properties in the brabender Viscoamylograph and the Rapid Visco-Analyzer. Cereal Chem. (1989) 66:499.

[31] Moraes ÉA Marineli RS Lenquiste SA Steel CJ de Menezes CB Queiroz VAV. Sorghum flour fractions: correlations among polysaccharides, phenolic compounds, antioxidant activity and glycemic index. Food Chem. (2015) 180:116–23. 10.1016/j.foodchem.2015.02.023 25766808

[32] Mpofu A Sapirstein HD Beta T. Genotype and environmental variation in phenolic content, phenolic acid composition, and antioxidant activity of hard spring wheat. J Agric Food Chem. (2006) 54:1265–70. 10.1021/jf052683d 16478246

[33] Guo N Tong T Ren N Tu Y Li B. Saponins from seeds of Genus *Camellia*: Phytochemistry and bioactivity. Phytochem. (2018) 149:42–55. 10.1016/j.phytochem.2018.02.002 29459215

[34] Arslan I Çelik A. Saponin-rich fractions (SRPs) from Soapwort show antioxidant and hemolytic activity. APCBEE Procedia. (2013) 7:103–8. 10.1016/j.apcbee.2013.08.019

[35] Avila-Roman J Soliz-Rueda JR Bravo FI Aragones G Suarez M Arola-Arnal A. Phenolic compounds and biological rhythms: who takes the lead? Trends Food Sci Technol. (2021) 113:77–85. 10.1016/j.tifs.2021.04.050

[36] Alamu EO Maziya-Dixon B Menkir A Irondi EA Olaofe O. Bioactive composition and free radical scavenging activity of fresh orange maize hybrids: impacts of genotype, maturity stages and processing methods. Frontiers Nutr. (2021) 8:640563. 10.3389/fnut.2021.640563 33718422 PMC7943467

[37] Awika JM Yang L Browning JD Faraj A. Comparative antioxidant, antiproliferative and phase II enzyme inducing potential of sorghum (*Sorghum bicolor*) varieties. LWT - Food Sci Technol. (2009) 42:1041–6. 10.1016/j.lwt.2009.02.003

[38] Beta T Hwang T. Influence of heat and moisture treatment on carotenoids, phenolic content, and antioxidant capacity of orange maize flour. Food Chem. (2018) 246:58–64. 10.1016/j.foodchem.2017.10.150 29291878

[39] Liu RH. Whole grain phytochemicals and health. J Cereal Sci. (2007) 46:207–19. 10.1016/j.jcs.2007.06.010

[40] Sánchez A Vázquez A. Bioactive peptides: a review. Food Qual Saf. (2017) 1:29–46. 10.1093/fqs/fyx006

[41] Giacco F Brownlee M. Oxidative stress and diabetic complications. Circ Res. (2010) 107:1058–70. 10.1161/CIRCRESAHA.110.223545 21030723 PMC2996922

[42] Min B Gu L McClung AM Bergman CJ. Chen MH. Free and bound total phenolic concentrations, antioxidant capacities, and profiles of proanthocyanidins and anthocyanins in whole grain rice (Oryza sativa L) of different bran colours. Food Chem. (2012) 133:715–22. 10.1016/j.foodchem.2012.01.079

[43] Gerits LR Pareyt B Delcour JA. Wheat starch swelling, gelatinization and pasting: Effects of enzymatic modification of wheat endogenous lipids. LWT - Food Sci Technol. (2015) 63:361–6. 10.1016/j.lwt.2015.02.035

[44] Elemosho AO Irondi EA Alamu EO Ajani EO Maziya-Dixon B Menkir A. Characterization of *Striga*-resistant yellow-orange maize hybrids for bioactive, carbohydrate, and pasting properties. Front Sustain Food Syst. (2020) 4:585865. 10.3389/fsufs.2020.585865

[45] Irondi EA Imam YT Ajani EO. Effect of *Brachystegia eurycoma* flour addition on the physicochemical properties of whole millet flour and the sensory attributes of its gluten-free bread. Acta Universitatis Cibiniensis Series E: Food Technol. (2021) 25:43–52. 10.2478/aucft-2021-0004

[46] Riley CK Wheatley AO Asemota HN. Isolation and characterization of starches from eight Dioscorea alata cultivars grown in Jamaica. Afr J Biotechnol. (2006) 5:1528–36.

[47] López-Barón N Gu Y Vasanthan T Hoover R. Plant proteins mitigate *in vitro* wheat starch digestibility. Food Hydrocolloids. (2017) 69:19*27. 10.1016/j.foodhyd.2017.01.015

[48] Ai Y Hasjim J Jane JL. Effects of lipids on enzymatic hydrolysis and physical properties of starch. Carbohydr Polym. (2013) 92:120–7. 10.1016/j.carbpol.2012.08.092 23218274

[49] Abiodun OA Akinoso R. Textural and sensory properties of trifoliate yam flour and stiff dough ‘amala’. J Food Sci Technol. (2015) 52:2894–901. 10.1007/s13197-014-1313-y 25892788 PMC4397313

[50] Hoover R Sailaya Y Sasulski FW. Characterization of starches from wild and long grain brown rice. Food Res Int. (1996) 29:99–107. 10.1016/0963-9969(96)00016-6

[51] Yu S Ma Y Menager L. Sun DW. Physicochemical properties of starch and flour from different rice cultivars. Food Bioprocess Tech. (2012) 5:626–37. 10.1007/s11947-010-0330-8

[52] Souare ML Traore L Husson F Lubbers S. Protein extractability and thermally induced gelation properties of African locust bean proteins (*Parkia biglobosa* Jacq. RBr). J Food Engr Technol. (2021) 10:19–27. 10.32732/jfet.2021.10.1.19

[53] Irondi EA Awoyale W Oboh G Boligon AA. Phenolics composition, antioxidant and pasting properties of high quality cassava flour substituted with *Brachystegia eurycoma* seed flour. Ann Univ Dunarea De Jos of Galati Fascicle Vi – Food Technol. (2019) 43:9–23. 10.35219/foodtechnology.2019.1.01

